# Sex determination of mummies through multi-elemental analysis of head hair using electrothermal vaporization coupled to inductively coupled plasma optical emission spectrometry†

**DOI:** 10.1039/d2ra05654b

**Published:** 2022-09-22

**Authors:** Margaret MacConnachie, Sarah Lu, Yangyang Wang, Jocelyn Williams, Diane Beauchemin

**Affiliations:** Queen's University, Department of Chemistry 90 Bader Lane Kingston ON K7L 2S8 Canada diane.beauchemin@queensu.ca; Trent University, Department of Anthropology 1600 West Bank Drive Peterborough ON K9L 0G2 Canada

## Abstract

Sex determination of human remains is of great archaeological significance, as it provides a more complete picture of social and familial structures within ancient societies. Typically performed through examination of bones in the pelvic region, accurate sex determination can be exceedingly challenging in the absence of a sufficiently preserved skeleton. Here, a method for sex determination in living humans, involving measurement of magnesium, strontium, sulfur, and zinc in head hair along with multivariate statistics, was applied for the first time to hair collected from 500 year-old mummies originating from Peru. Hair samples were washed in doubly deionized water, dried, and ground prior to analysis *via* electrothermal vaporization coupled to inductively coupled plasma optical emission spectrometry; only 2 mg of hair is required for analysis. Point-by-point internal standardization was performed with Ar i 430.010 nm to compensate for sample loading effects on the plasma. Peak areas were integrated and mass corrected before being used in combination with multivariate analysis. Although principal component analysis provided insufficient separation between the sexes, linear discriminant analysis (LDA) was highly effective for sex determination. Using mummy hair as the LDA model enabled accurate sex prediction of the mummies, showing that, despite the age of the hair, the samples still contain the necessary elemental information for sex determination. For accurate sex determination of mummies using hair collected from living humans, magnesium had to be replaced by sodium due to significant differences in dietary habits. With this simple modification, hair from living humans in North America could be used to successfully predict the sex of individuals who lived more than 500 years ago in Peru. This work paves the way for broader use of non-skeletal sex determination methods within the field of archaeology, filling a significant gap.

## Abstract 2

Multi-elemental analysis of hair from living humans allows for accurate, non-skeletal sex determination of 500 year-old mummified individuals.

## Graphical abstract

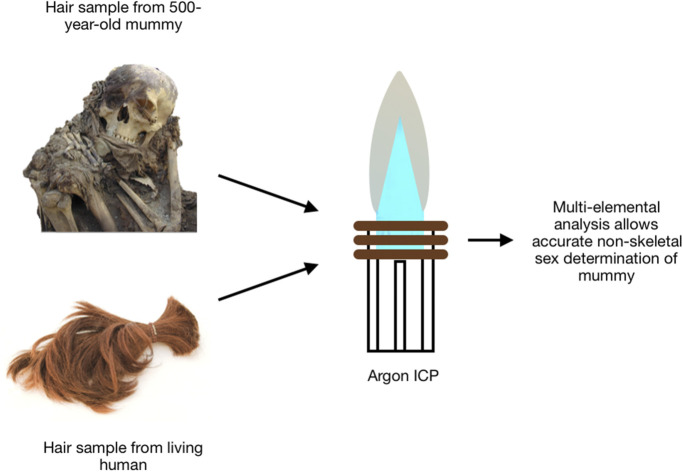

## Introduction

Accurate sex determination of human remains is a vital part of archaeological and anthropological analyses because it provides a deeper understanding of the social and biological structures of ancient societies,^1^ and is useful in providing a complete biological profile of an individual.^2^ Sex determination is often one of the first steps in an archaeological investigation because sex ratios give important insight into the nature of the culture, and shape the way that researchers understand relationships in ancient societies.^3^ Accurate sex determination can also aid in determining other parameters, such as stature, age, and occupation.^2^

Currently, the most widely accepted method for sex determination in adults is based on the examination of morphological features of the *os coxae*.^4–7^ This method is not only highly accurate,^6,7^ but is also reliable because it can be successfully applied to any skeleton, regardless of geographical origin or age of the remains.^8^ Indeed, the sexual dimorphism in the pelvic region allows for accurate sex determination across populations and has been observed in the last 100 000 years of human history.^8^ Other methods of sex determination have also been developed for additional skeletal elements, including the skull,^4^ long bones,^9^ proximal ulna (forearm),^6^ and bones of the hands and feet.^10^ However, these methods are not considered to be population independent.

In any case, relying on bones for sex determination can present significant problems, as they can easily be fragmented, broken, and poorly preserved.^2^ In the absence of sufficiently preserved skeletal remains, accurate sex determination is extremely challenging.^4^ While deoxyribonucleic acid (DNA) analysis of ancient bones may be possible, and does not require complete remains, it presents several challenges. In addition to requiring that the DNA be sufficiently preserved, there are high chances of sample contamination, both from environmental factors, and the researchers performing excavation and analysis.^11^

In a proof-of-concept paper, Huang and Beauchemin showed that a living individual's sex could be predicted with a high degree of accuracy from the relative distributions of Mg, S, Sr, and Zn in head hair.^12^ This was achieved through direct multi-elemental analysis of head hair by electrothermal vaporization (ETV) into inductively coupled plasma optical emission spectrometry (ICPOES) in combination with multivariate statistics using principal component analysis (PCA) and linear discriminant analysis (LDA) for dimensionality reduction.^12^ Because hair is not remodelled, it contains a permanent record of elements ingested by an individual,^13^ which is harnessed by this ETV-ICPOES method. Most endogenously absorbed elements originate from the secretions of four sweat glands: eccrine, apocrine, sebaceous, and epidermis.^14^ Females tend to have higher concentrations of Ag, Sr, and Mg, but lower concentrations of Mn, Mo, Sn, K, Pb^15^ than males because males typically have enlarged sweat glands, leading to increased secretions of certain elements.^16^ The ETV-ICPOES method has so far solely been applied to hair from living individuals.

With ETV, the direct analysis of solids, liquids, and slurries requires only 1–5 mg of sample,^17^ which is useful for the analysis of rare and precious archaeological materials. In allowing the direct analysis of solid samples, sample digestion is no longer required, reducing sample contamination and the overall analysis time, and enabling analysis with smaller sample amounts. ETV has allowed for accurate analysis of hair in several forensic applications, paving the way for the technique to be used to study hair in an archaeological context. When coupled to inductively coupled plasma mass spectrometry (ICPMS), ETV has been used in the multi-elemental analysis of hair,^18^ detection of Hg within the hair shaft,^19^ and, in a case involving Tl poisoning, measurement of the distribution of Tl along the hair shaft, allowing for retroactive determination of the time of exposure.^20^ Accurate multi-elemental analysis of hair was also carried out by ETV-ICPOES using slurry sampling,^21^ in addition to the above-mentioned solid sampling method for sex determination.^12^

Several other analytical methods for the analysis of hair (such as ICPMS^18–20,22,23^ and atomic absorption spectrophotometry^24^) require digestion of several hundred mg^25–27^ to 1 g of hair,^22–24^ which is not feasible for rare archaeological samples such as mummy hair. In contrast, solid sampling ETV requires only 2 mg of hair per replicate. ICPOES is often coupled to ETV when solid sampling is being performed due to the inherent robustness of the technique.

The objectives of this work were to apply the ETV-ICPOES method for sex determination to hair samples originating from 500 year-old mummified human remains from Puruchuco-Huaquerones, which is a Late Horizon (A. D. 1476–1532)^13^ archaeological zone located in the middle of the Rimac valley on the central coast of Peru. This zone encompasses several cemeteries, a site museum, and several architectural components.^28^ Excavations of Puruchuco-Huaquerones between 1998 and 2001 revealed 1286 burials, where individuals underwent spontaneous mummification due to the extreme aridity of the region.^13^ Under arid conditions, hair becomes relatively resistant to diagenesis, and can be preserved for hundreds of years, making it especially valuable in archaeological investigations.^13^ The present study includes testing of hair sample washing methods in addition to assessing the viability of multi-elemental analysis of the centuries-old hair samples, and using living humans to determine the sex of the mummified individuals. To the best knowledge of the authors, this is the first time that the analysis of mummy hair for accurate sex determination is reported using ETV-ICPOES.

## Methods and materials

### Instrumentation

Analysis was performed using a lateral plasma view ARCOS ICPOES instrument (SPECTRO Analytical Instruments, Kleve, Germany), coupled to an ETV system (ETV 4000C, Spectral Systems, Furstenfeldbruck, Germany). Instrumental parameters for ETV-ICPOES are shown in Table 1. Samples were weighed into pyrolytically coated graphite boats (Meinhard, Golden, CO, USA), and manually inserted into the ETV furnace using tweezers. A 1 m long Teflon tube connected the furnace to the ICP torch. Transient signals were recorded using Smart Analyzer Vision software, and raw data were exported to Microsoft Excel for processing.

Instrumental operating conditions for ETV-ICPOES, based on conditions from Huang and Beauchemin.^12^

**Table:** 

Parameter	Setting
Ar plasma gas flow rate (L min^−1^)	14
Ar auxiliary gas flow rate (L min^−1^)	3.0
RF power (kW)	1.4
Signal scan mode	Transient
Integration time (ms)	10
Sampling rate (Hz)	0.5
Observation height (mm)	10
Ar bypass gas flow rate (L min^−1^)	0.3
Ar carrier gas flow rate (L min^−1^)	0.3
CF_4_ reaction gas flow rate (mL min^−1^)	4.00

**Table:** 

ETV temperature program – analysis of samples		
Step	Temperature (°C)	Time (s)
Initial temperature	21	—
Pyrolysis	500	20
Cooling	No heating	15
Vaporization	2200	30
Cooling	No heating	20

**Table:** 

ETV temperature program – cleaning		
Step	Temperature (°C)	Time (s)
Initial temperature	21	1
Cleaning	2300	45
Cooling	No heating	15

The following emission lines (in nm) were monitored: Al i (309.271), Ar i (430.010), Ca ii (393.366), Ce ii (393.373), Fe ii (238.204), Mg ii (280.270), Na i (588.995), S i (180.731), S i (182.034), Sr ii (394.080), Sr ii (407.771), Ti i (334.187), Zr ii (343.823), Zn ii (206.200). Analyte emission lines were selected based on maximum sensitivity and freedom from spectroscopic interferences. The Ar emission line was used for internal standardization, to compensate for the reproducible signal suppression that occurs during vaporization from sample loading effects on the plasma.

### Reagents and gases

Argon (99.996% purity, liquid in Dewar, MEGS Specialty Gases, Ottawa, ON, Canada) was used for the plasma, auxiliary, bypass, and carrier gases. CF_4_ reaction gas (MEGS Gases, Ottawa, ON, Canada) was added to the ETV carrier gas to increase analyte volatility. Doubly deionized water (DDW) (18.2 MΩ cm^−1^) was provided by an Arium Ori UV/DI water purification system (Sartorius Stedim Biotech, Göttingen, Germany). Hexane (Caledon Laboratories Ltd, Georgetown, Ontario, Canada) and acetone (ACS grade; Fisher Scientific, Ottawa, ON, Canada) were also used to wash hair.

### Sample preparation

**Fig. 1:**
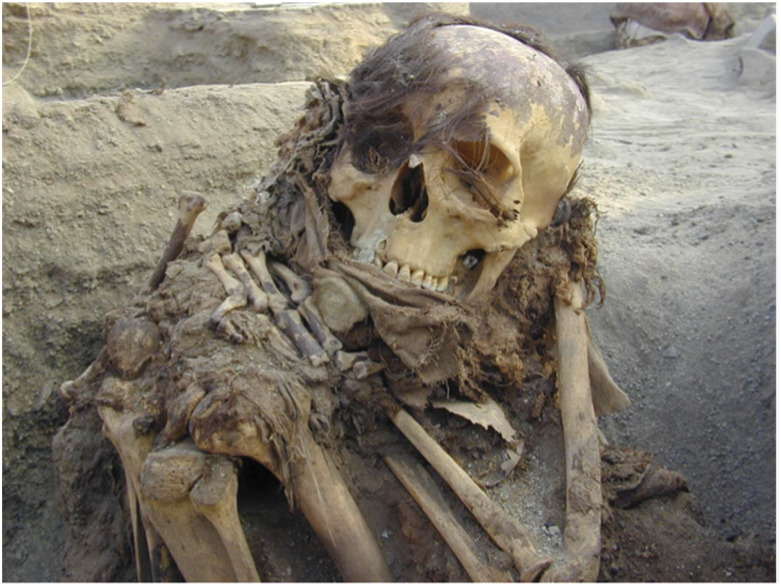
Sample 28 *in situ* at the excavation site at Puruchuco-Huaquerones, Peru.

Eight human hair samples and nine mummy hair samples were used in this study, sample names and classifications are shown in Table 2. All nine mummy hair samples originate from mummified individuals from Puruchuco-Huaquerones, Peru (Fig. 1). The hair was collected directly from the mummified heads of individuals who had abundant hair (a minimum of 20 strands). A scalpel (cleaned with alcohol) was used to excise a portion of the scalp with the hair embedded. If the mummy had been exposed or was poorly preserved, the hair would no longer adhere to the scalp but be lying next to the scalp in the wrappings surrounding the head. In this case, it was collected by hand and the scalp end wrapped in tinfoil and then taped secure to orient the strand. In all circumstances, the hair was still associated with the skull, and it was not taken from any other location or materials on the body. All eight human hair samples were collected in Kingston, Ontario, Canada from adults of varying ethnicities. All of the hair samples collected were roughly the same length (10–15 cm).

**Table:** Hair samples used in this study. All hair samples originate from adults

Sample name	Sample group	Sex	Sample name	Sample group	Sex
AK	Human	Male	21	Mummy	Male
TS		Male	58		Male
AC		Male	32		Male
KB		Male	50		Male
YW		Female	33		Female
DC		Female	41		Female
EG		Female	28		Female
CC		Female	14		Female
			19		Female

Each sample was washed with three 30 mL aliquots of DDW, then dried and ground into a fine powder using a mortar and pestle. Because at least 100 mg^31^ is considered a representative sample in hair analysis, the entirety of the length, *i.e.* 150–200 mg of hair, was ground for each sample to ensure that the 2 mg aliquots would be homogenous and representative. This effectively mitigated longitudinal variations, which require portioning hair into typically 3–10 mm segments to be visible.^20,32–34^

### Data processing

**Fig. 2:**
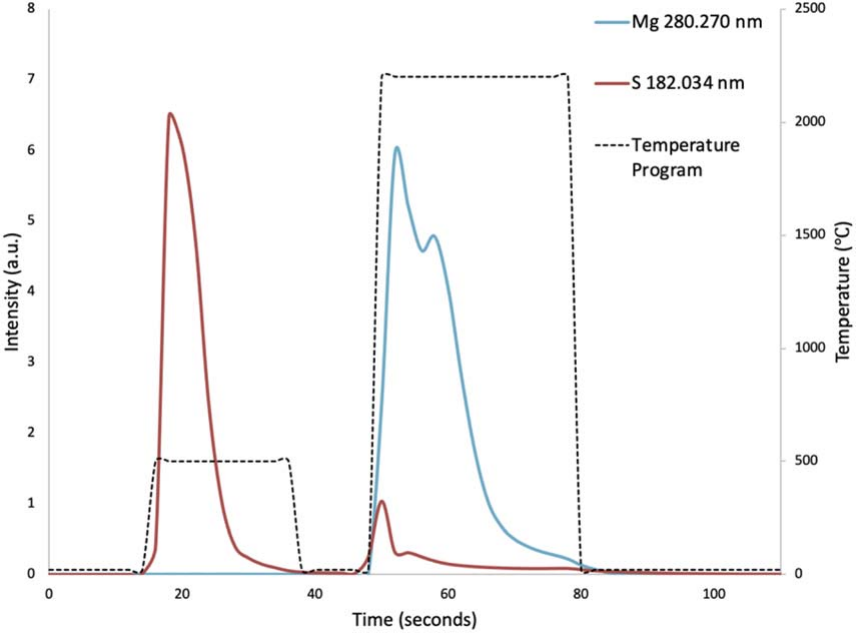
Vaporization profiles of sulphur and magnesium, showing the premature vaporization of sulphur during the pyrolysis step.

To compensate for sample loading effects on the plasma, point-by-point internal standardization was performed with the Ar 430.010 nm emission line. The peak area of the transient signal of each sample was then integrated during the vaporization step, and the peak areas were mass corrected. Due to the low vaporization temperature of sulphur, the majority of the sulphur contained in the hair samples was vaporized prematurely during the pyrolysis step (Fig. 2). The peak area of the transient signal of sulphur was thus integrated over the pyrolysis step, in addition to the vaporization step. Multivariate analysis was performed using Minitab software (Version 19.2020.2.0), purchased *via* OnTheHub.

### Analysis procedure

Ten blank signals were collected, through analysis of empty graphite boats. The peak area of each of the transient signals was integrated during vaporization, and the average of these 10 values was used for blank subtraction. Three replicates of approximately 2 mg of each sample were weighed and placed in graphite boats using tweezers, before being inserted into the ETV furnace.

### Investigation of washing methods

Three different washing methods were tested to assess the viability and reproducibility of each method. The first is the method described by Huang and Beauchemin using three 30 mL aliquots of hexanes, followed by three 30 mL aliquots of DDW.^12^ The second involves washing in a 25 mL aliquot of DDW, followed by a 25 mL aliquot of reagent-grade acetone, then a final wash in a 25 mL aliquot of DDW.^29^ This method was adapted from the International Atomic Energy Agency (IAEA) protocol for hair washing.^30^ The final was adapted from a hair washing method described by Williams *et al.*, which involves washing in three 30 mL aliquots of DDW.^13^ One sample of mummy hair was chosen randomly and washed using the three described methods.

## Results and discussion

### Comparison of washing methods

Typically, washing methods involve water, organic solvents, or surfactants such as Triton-X,^31^ or combinations of the three. Huang and Beauchemin used portions of hexanes followed by portions of doubly deionized water (DDW) to wash hair prior to analysis,^12^ to remove external dirt or build up that may potentially alter the results. Given that exogenous trace elements may be incorporated into human hair in such a way that they cannot be washed off without removing some of the endogenous trace elements,^14^ a minimal washing procedure is preferable. Indeed, the IAEA washing method can remove endogenous trace elements, in addition to the exogenous elements.^35^ Sonication of the hair during the washing procedure can result in the loss of endogenous elements.^36^ The use of compounds such as ethylenediaminetetraacetic acid in hair washing resulted in the loss of endogenous elements, as well as exogenous elements.^37^ Aqueous solutions such as methanol are typically avoided for washing as they cause swelling and damage in the hair cuticle, resulting in the removal of endogenous elements.^38^ This was of particular concern for the mummified hair due to the extreme brittleness resulting from moisture loss during the mummification process. In the case of mummy hair, heavy build up (from hair products, for example) is not a concern, but external dirt presents the largest source for potential contamination. Additionally, as all of the mummies in this study underwent spontaneous mummification,^13^ external contamination from embalming materials is not an issue.

Three different washing methods were tested: washing in hexanes and DDW, washing in acetone and DDW, and washing only in DDW. The washing method caused no significant difference in the measured concentrations of the monitored elements at the 95% confidence level using one-way analysis of variance (*F* scores ranging from 0.43–4.48, *F*_crit_ = 5.14). However, washing with only DDW produced an average relative standard deviation (RSD) of 6.6 ± 3.2%, compared to average RSDs of 13 ± 11% and 15.9 ± 6.9% when also including washes with hexane and acetone, respectively. Hence, washing with only DDW was used thereafter. In addition to improving precision, it eliminates organic solvent consumption and waste. This agrees with the finding by Raposo *et al.* that ultra-pure water effectively cleansed surface dirt and grease without decreasing endogenous element concentrations.^35^ The above RSDs are typical for solid sample analysis by ETV. They did not prevent LDA from being used successfully for accurate sample discrimination and classification in several other cases with comparable RSDs.^12,39,40^

### Mummy hair as the training set

To assess whether mummy hair contains the necessary elemental information for sex determination, the ETV-ICPOES method with the original set of elements as predictors (Mg, S, Sr, and Zn)^12^ was applied by using mummy hair samples as the training set to predict the sex of other mummies. Sample classification with PCA (Fig. S1†) was unsuccessful at accurately separating the samples according to sex. Unlike PCA, LDA is a supervised algorithm that takes group classifications into account, which made it more effective in sample discrimination in both hair samples,^12^ and automotive paint samples.^39,40^ To test the method using LDA, the mummy hair samples had to be treated as blind samples. Eight samples were used as the training set and the ninth sample was left out and used as the unknown, ensuring that the unknown sample was a true blind sample. Each of the nine samples were treated as an unknown. This is known as ‘leave-one-out cross validation’ and is considered to be the least biased way of testing an LDA training set.^41^

As can be seen in Table 3, LDA was very effective in accurately predicting the sex of all nine samples, and provided accurate predictions with 100% probability in all cases (complete LDA predictions in Table S1†). Not only do these results further validate the method developed by Huang and Beauchemin,^12^ but they show that, even though the hair samples are over 500 years old and were not intentionally preserved,^13^ they still contain the necessary elemental information for accurate sex determination. Clearly, the hair samples were sufficiently resistant to diagenesis to allow their use in this method. However, sex determination of mummified individuals using other mummified individuals in the training set would present a significant barrier to the practical use of this method by requiring the need to have several mummified individuals, who have been accurately sexed using skeletal features, from the same geographic area. The aim of this work is to predict the sex of mummified individuals using hair samples from living humans.

**Table:** LDA classification results for the mummy hair samples, using mummy hair and human hair samples for the training set with Mg, S, Sr, and Zn as predictor elements

Training set	Sample classification	# samples predicted as male	# samples predicted as female	% correctly predicted
Mummy	Male	4	0	100%
	Female	0	5	100%
Human	Male	2	2	50%
	Female	0	5	100%

### Human hair as the training set

When eight human hair samples were used to predict the sex of the nine mummy hair samples, with Mg, S, Sr, and Zn as predictor elements,^12^ PCA remained ineffective in accurately classifying the samples (Fig. S2†). The PCA score plots for the other eight mummy samples (not shown) yielded similar results. In fact, in several score plots, even the human samples were not accurately separated. Using LDA and the eight human hair samples in the training set, each mummy hair sample was treated as a blind, with the results in Table 3 (complete LDA predictions in Table S2†). While all of the female mummy samples were predicted correctly with 100% probability, half of the male samples were also predicted as females, with 100% probability.

### Exclusion of magnesium

**Fig. 3:**
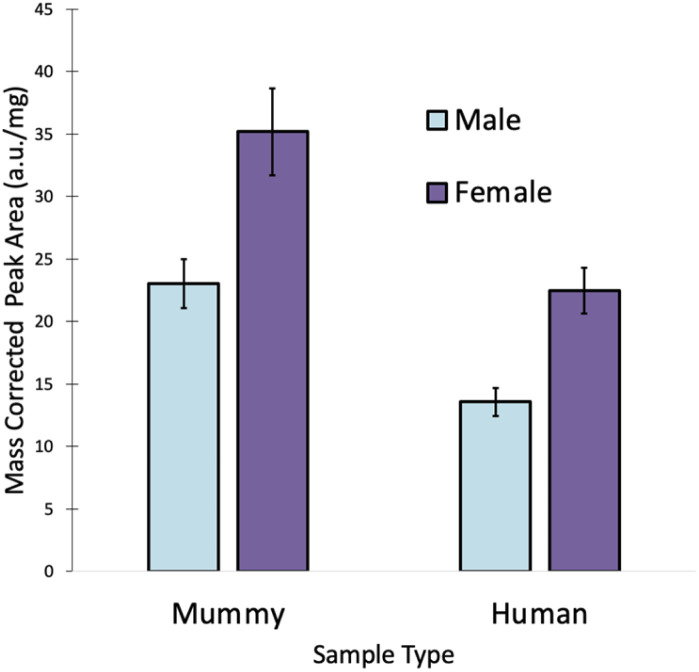
Comparison of Mg levels in human and mummy hair samples (*n* = 12).

In order to determine why some of the male samples were predicted as females, the levels of all four elements were examined. Fig. 3 shows that there is a noticeable difference in the amount of Mg in the human and mummy hair samples. No such difference was visible for S, Sr, and Zn (not shown), which had comparable levels in the human and mummy hair samples. As expected, females tend to have higher Mg levels than males in both mummy and human hair samples.^15^ However, a Student's *t*-test at the 95% confidence level revealed that the male mummy hair contained significantly more Mg than the male human hair (*p* = 0.0002), and that the female mummy hair contained significantly more Mg than the female human hair (*p* = 0.0003).

In fact, the female human hair samples and the male mummy hair samples have Mg levels that are not significantly different from each other (*p* = 0.69). This resulted from the diet of the mummified individuals, which consisted primarily of maize, beans, sweet potatoes, marine and riverine fish, and marine invertebrates,^13^ all foods with high Mg concentrations. While the community at Puruchuco-Huaquerones did eat a variety of foods including terrestrial fauna, such as camelids, guinea pigs, and dogs,^13,28^ their dietary staples were Mg-rich foods. Despite being a very strong predictor of sex in living individuals,^12^ the large discrepancy between Mg levels in the human and mummy hair samples caused some inaccurate predictions. Hence, for accurate predictions by LDA, Mg must be removed from the method.

### Selection of another predictor element

Several elements (Al, Cr, Fe, Ti, Zr) showed strong loading values in PCA loadings plots (not shown) but their levels were significantly different in at least one of the sexes (*p* values range from 0.0001 to 0.0467). As a result, replacing Mg by each of these elements in LDA produced mispredictions, or inconclusive predictions (posterior probability of less than 0.7, known as a ‘in-doubt unit’ or a ‘fence rider’), as exemplified with Ti in Table 4. Hence, in addition to being strong predictors of sex, the selected elements must be present at levels that are comparable between the training set and sample sets in order for accurate sex predictions to be possible.

**Table:** LDA classification results of the mummy hair samples, with the human hair samples used as the training set

Elements used as predictors	Sample classification	# samples predicted as male	# samples predicted as female	% correctly predicted
S, Sr, Ti, and Zn	Male	4	0	100%
	Female	2	3	60%
Na, S, Sr, and Zn	Male	4	0	100%
	Female	0	5	100%

Because Na showed not only strong loadings, but crucially had comparable levels in both the human and mummy hair samples sets, it was selected to replace Mg as a predictor in further trials. PCA score plots (Fig. S3†) resulted in only three samples being correctly classified. With LDA, as can be seen in Table 4, the sex of all nine mummy hair samples were accurately predicted with 100% probability using human hair samples with considerable differences in both sample age and geographic origin (complete LDA predictions in Table S3†).

## Conclusions

This work demonstrated that hair from living humans in North America could be used to accurately predict the sex of individuals who lived more than 500 years ago in Peru, with as little as 2 mg of hair needed for analysis when employing ETV for sample introduction into ICPOES. No quantification is required with ETV-ICPOES, as prediction relies on the relative amounts of the elements within the hair samples. This approach not only simplifies the analysis and shortens the overall analysis time compared to methods requiring sample dissolution, but it also eliminates the need for matrix-matched solid certified reference materials.

Washing the hair samples with DDW proved to be effective, and more reproducible than other washing methods involving harsh organic solvents. PCA alone was insufficient in accurately separating all of the male and female samples. However, LDA was able to accurately group all samples with 100% probability after replacing Mg by Na as predictor element in the method. The large ethnic variation between the living human samples and the mummified individuals further confirms the results obtained by Huang and Beauchemin, which indicate that sex and ethnicity are independent of each other. This work highlights the potential for hair analysis within the field of archaeology and archaeological sciences. Even hair that was not intentionally preserved is resistant enough to diagenesis to hold the elemental information necessary for sex determination. Importantly, this work paves the way for broader use of non-skeletal sex determination methods, filling a significant gap within the field of archaeology.

Future work will involve applying this method to hair collected from mummified children. All of the currently established methods for sex determination of skeletal remains rely on sexual dimorphism, something that is not present in immature skeletons. Currently, there are no methods to determine sex accurately and reliably in subadult human skeletal remains.^42^ If the described method is able to be successful in accurately predicting the sex of mummified children, it would aid in filling a significant gap within the field of archaeology. Future work may also include applying this method to bone tissue. Bone is a living tissue that is constantly being remodelled and renewed; new bone is deposited, and old bone is dissolved.^28^ As the tissue is continually formed throughout life, it holds a record of trace elements that remains even after the tissue is destroyed.^43^ Bone tissue represents the average diet over a period of ‘bone turnover’ (the amount of time it takes for the entire skeleton to be renewed), typically 10–25 years;^28^ this elemental information may prove useful in sex determination. Hair and nails are much rarer than skeletal remains in archaeological contexts,^13^ so application of this method to bone fragments may be both beneficial and practical.

## Author contributions

Margaret MacConnachie: data curation, formal analysis, software, supervision, validation, visualization, writing (original draft), and writing (review & editing). Sarah Lu: investigation and writing (review & editing). Yangyang Wang: investigation and writing (review & editing). Jocelyn Williams: conceptualization, resources, and writing (review & editing). Diane Beauchemin: conceptualization, funding acquisition, methodology, project administration, resources, supervision, visualization, and writing (review & editing).

## Conflicts of interest

There are no conflicts to declare.

## Supplementary Material

RA-012-D2RA05654B-s001
