# Supplementary material for: Sex determination of mummies through multi-elemental analysis of head hair using electrothermal vaporization coupled to inductively coupled plasma optical emission spectrometry
Source: RSC Adv. 2022 Sep 22;12(42):27064–71. doi: 10.1039/d2ra05654b (PMC9494207; doi:10.1039/d2ra05654b)
Supplement: RA-012-D2RA05654B-s001 [file RA-012-D2RA05654B-s001.pdf]

Electronic Supplementary Material (ESI) for RSC Advances.
This journal is © The Royal Society of Chemistry 2022

Supplementary Material for:

Sex Determination of Mummies through Multi-Elemental Analysis of Head Hair using Electrothermal Vaporization Coupled to Inductively Coupled Plasma Optical Emission Spectrometry

by M. MacConnachie, S. Lu, Y. Wang, J. Williams, and D. Beauchemin

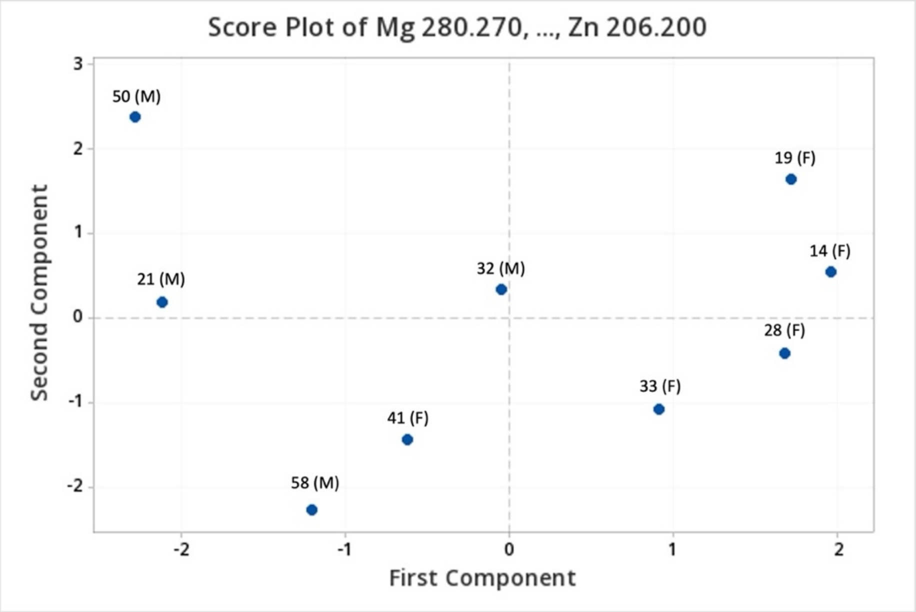

Fig. S1 PCA score plot using Mg, S, Sr, and Zn as predictors. Only mummy hair samples have been included in the plot.

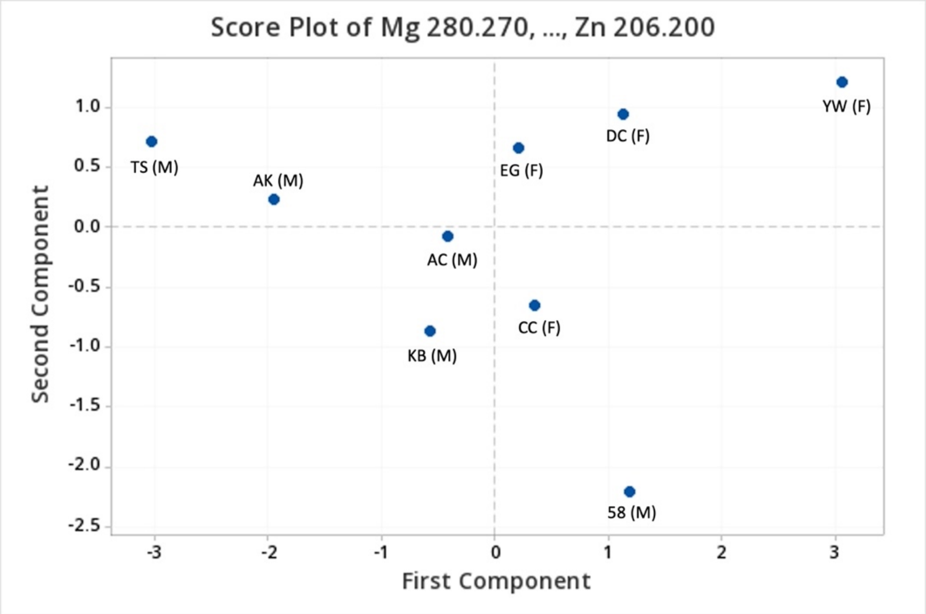

Fig. S2 PCA score plot using Mg, S, Sr, and Zn as predictors. Eight human samples and one mummy sample (58, male) have been included in the plot.

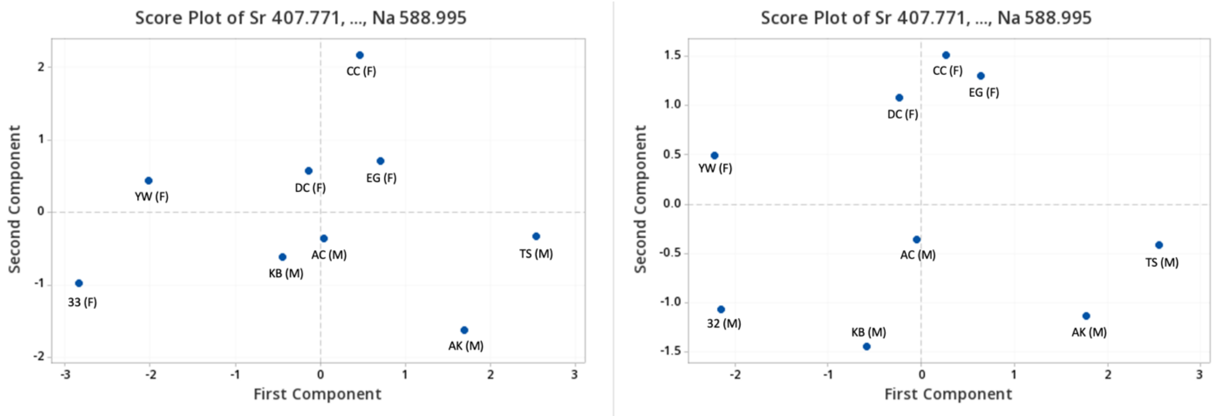

Fig. S3 PCA score plots using Na, S, Sr, and Zn as predictors. Each score plot contains all eight human samples, and one mummy sample. The left score plot contains sample 33 (female), and the right score plot contains sample 32 (male).

Table S1 Predictions by LDA for the nine mummy hair samples, using the other mummy hair samples as the training set, with Mg, S, Sr, Zn as predictors.

Sample	Known Group	Probabilities		Predicted Group
21	Male	Male	1.000	Male
		Female	0.000	
58	Male	Male	1.000	Male
		Female	0.000	
32	Male	Male	1.000	Male
		Female	0.000	
50	Male	Male	1.000	Male
		Female	0.000	
33	Female	Male	0.000	Female
		Female	1.000	
41	Female	Male	0.000	Female
		Female	1.000	
28	Female	Male	0.000	Female
		Female	1.000	
14	Female	Male	0.000	Female
		Female	1.000	
19	Female	Male	0.000	Female
		Female	1.000	

Table S2 Predictions by LDA for the nine mummy hair samples, using the human hair samples as the training set, with Mg, S, Sr, Zn as predictors

Sample	Known Group	Probabilities		Predicted Group
21	Male	Male	1.000	Male
		Female	0.000	
58	Male	Male	0.000	Female
		Female	1.000	
32	Male	Male	0.000	Female
		Female	1.000	
50	Male	Male	1.000	Male
		Female	0.000	
33	Female	Male	0.000	Female
		Female	1.000	
41	Female	Male	0.000	Female
		Female	1.000	
28	Female	Male	0.000	Female
		Female	1.000	
14	Female	Male	0.000	Female
		Female	1.000	
19	Female	Male	0.000	Female
		Female	1.000	

Table S3 Predictions by LDA for the nine mummy hair samples, using the human hair samples as the training set, with Na, S, Sr, and Zn as predictors

Sample	Known Group	Probabilities		Predicted Group
21	Male	Male	1.000	Male
		Female	0.000	
58	Male	Male	1.000	Male
		Female	0.000	
32	Male	Male	1.000	Male
		Female	0.000	
50	Male	Male	1.000	Male
		Female	0.000	
33	Female	Male	0.000	Female
		Female	1.000	
41	Female	Male	0.000	Female
		Female	1.000	
28	Female	Male	0.000	Female
		Female	1.000	
14	Female	Male	0.000	Female
		Female	1.000	
19	Female	Male	0.000	Female
		Female	1.000	
